# Case of Secondary Hemorrhage, Occurring on the Fourth Day after Delivery, the Result of the Rupture of a Bloody Tumor in the Cervix Uteri

**Published:** 1851-10

**Authors:** George Johnston

**Affiliations:** Assistant Physician to the Lying-in Hospital


### RECORD OF MEDICAL SCIENCE.

### OBSTETRICS.

*Case of Secondary Hemorrhage, occurring on the fourth day after Delivery, the Result of the Rupture of a bloody Tumor in the Cervix Uteri.* By George Johnston, M. D., Assistant Physician to the Lying-in Hospital.—M. H., aged 35, a strong, robust country-woman, was admitted into the Rotundo Lying-in Hospital, late on the night of the 21st September, 1850, labor pains having unexpectedly set in about three hours before. She states that it is her seventh pregnancy, that she has five children living, one having been dead-born at the full period, for which she could assign no cause; had always easy confinements; that considering herself to be only in the eighth month, she had travelled up on foot from the country, a distance of eighty miles, about four or five days previously, for the purpose of arranging pecuniary matters, calculating that she would be able to return home in sufficient time to be confined. During her journey, which occupied upwards of two days, she suffered much from fatigue, and exposure to cold and wet, which, together with her distress of mind, occasioned by the purport of her mission, all tended to bring on premature labor. The fœtal heart could not be heard, nor had she felt the motion of the child since her arrival in town. On examination *per vaginam,* the os uteri was found to be about the size of a crown piece, breech presenting, membranes unruptured, pains apparently not of much strength. However, after a short and easy labor of about four hours' duration, the child was born with the membranes entire; on rupturing them, it was discovered to be a female of about eight months, and bore evident signs of having been dead for some time. The placenta was expelled in about ten minutes afterwards; no hemorrhage nor any untoward symptom supervened, and everything went on favorably for the first three days. The milk was secreted on the second day. At the morning visit of the 25th (her fourth day) she expressed herself quite strong; a cough which she had contracted on her journey, much better; no complaint, nor any uneasiness whatever. At half-past 1, P. M., the nurse called me in a great hurry, stating that the patient had been suddenly attacked with violent hemorrhage. On inquiry I found that she had not been out of bed, nor had she been using any exertion. On reaching the bed-side (which was in less than a minute after hearing the report, and certainly not more than three from the first gush of blood), I found her lying on her back, countenance perfectly blanched, and expressive of great anxiety, which, with her neck, hands, and arms, was bathed in cold clammy perspiration. No pulse could be felt at the wrist; and the bed inundated with blood, which was still flowing rapidly from the vagina. The pillows were at once removed from underneath her head, thus placing her completely in the horizontal position; firm pressure was made over the uterus (which was found well contracted;) cold applied externally, and a stream of cold water was injected into the vagina and rectum. She was given three ounces of wine, with half a drachm of ergot of rye; at the same time a current of cold air was allowed to pass across her face; all of which had the effect of restraining the great flow, but not of completely checking the hemorrhage, for a slight trickling still continued. However, having restored somewhat the temperature of the body, by means of hot jars to the feet and warm blankets to the extremities, by stimulants (brandy) frequently repeated, beef-tea, &c., we were enabled once more to feel the pulse at the wrist; the color of the countenance returned slightly, giving us hopes that the worst had been overcome. The ergot was repeated, with forty drops of the acetum opii in an ounce of brandy and water, and pressure was maintained over the uterus by the hand for nearly half an hour. When about re-applying the binder, another sudden flow of blood took place, the hand being still on the uterus, and with it the expulsion of a large coagulum. She again became pulseless, and fainted, cold clammy perspiration once more bedewing the surface of her face, neck, and arms; this was succeeded by extreme restlessness with great anxiety, and the respiration became labored and gasping. Stimulants were again attempted to be administered, but they were with difficulty swallowed, and shortly after rejected; and she too soon gave evidence that all our efforts were unavailing, as she rapidly sank, just one hour and a half after the first attack of hemorrhage.

*Autopsy.*—The thoracic and abdominal viscera were found quite healthy, but pale and bloodless. The uterus was well contracted down in the pelvis. On closer inspection, just beneath the peritoneal covering, in the left iliac fossa, a tumor of moderately firm consistence was seen, about the size of a large walnut, having an ecchymosed appearance, which extended also some little distance on the outer side: this tumor was firmly attached to the lower part of the uterus. On removing the latter with its appendages, and laying it open from the os to the fundus, cutting in the mesial line through the anterior wall, the muscular structure was found to be quite healthy, and that portion of the inner surface which had been occupied by the placenta was well plugged with a dark and firm clot, portions of which were seen entering the mouths of the uterine sinuses, thus proving that they had not been the source of the hemorrhage. On the left side of the cervix, about one inch from the os uteri, was observed a ragged, sloughy-looking opening, the edges of which were very irregular, and of a black ash-grey color. This opening, which was large enough to admit two fingers easily, communicated with a cavity the size of a small orange; it seemed to be formed in the substance of the cervix, and its external wall was found to be the projecting tumor before mentioned, as seen from the outside. On laying open this cavity, and washing away some loose clots (but carefully observing that there were no laminated coagula,) the lining membrane was found rugous, of a firm consistence, and resembling very much in appearance the mucous membrane of the vagina. Opening into this sac were seen the mouths of five or six blood-vessels large enough to admit a small bougie. Upon introducing a blowpipe into these open mouths, and inflating them, it was clearly demonstrated that they communicated with the uterine sinuses, for bubbles of air could be driven out of the vessels at the edge of the uterus where we had divided it, and with care and delicate manipulation pieces of bougie could be passed for a distance of nearly three or four inches along these ducts, which ran in various directions, some longitudinally, some transversely, and could even be protruded in one or two instances through their cut openings.

This case was read before the Obstetrical Society on the first night of its meeting this session, when it was considered by some of the members present to be a thrombus or bloody tumor occurring during labor. This opinion, so far, at least, as the former part of it is concerned, has been corroborated by Dr. Carte, the Curator of the College of Surgeons' Museum, who was kind enough to make a careful examination of the sac; but at what period the formation of it took place remains doubtful, for it hardly could have occurred during labor, which was extremely easy, as is evidenced both by the short time it occupied, and the manner in which the fœtus was expelled, viz., in the membranes. It is, therefore, a case of peculiar interest, as well as an instance of one of the many casualties the obstetrician is liable to encounter in his course of practice, by which his reputation might be placed at stake and his character deeply involved; distressing, too, from its being almost, if not wholly, beyond his control, and which the vigilance of his most watchful anxiety would be unable to foresee.—*Dublin Quarterly Journal.*

